# Novel electrochemical redox-active species: one-step synthesis of polyaniline derivative-Au/Pd and its application for multiplexed immunoassay

**DOI:** 10.1038/srep16855

**Published:** 2015-11-18

**Authors:** Liyuan Wang, Feng Feng, Zhanfang Ma

**Affiliations:** 1 Department of Chemistry, Capital Normal University, Beijing 100048, China

## Abstract

Electrochemical redox-active species play crucial role in electrochemically multiplexed immunoassays. A one-pot method for synthesizing four kinds of new electrochemical redox-active species was reported using HAuCl_4_ and Na_2_PdCl_4_ as dual oxidating agents and aniline derivatives as monomers. The synthesized polyaniline derivative-Au/Pd composites, namely poly(N-methyl-o-benzenediamine)-Au/Pd, poly(N-phenyl-o-phenylenediamine)-Au/Pd, poly(N-phenyl-p-phenylenediamine)-Au/Pd and poly(3,3’,5,5’-tetramethylbenzidine)-Au/Pd, exhibited electrochemical redox activity at −0.65 V, −0.3 V, 0.12 V, and 0.5 V, respectively. Meanwhile, these composites showed high H_2_O_2_ electrocatalytic activity because of the presence of Au/Pd. The as-prepared composites were used as electrochemical immunoprobes in simultaneous detection of four tumor biomarkers (carcinoembryonic antigen (CEA), carbohydrate antigen 19-9 (CA199), carbohydrate antigen 72-4 (CA724), and alpha fetoprotein (AFP)). This immunoassay shed light on potential applications in simultaneous gastric cancer (related biomarkers: CEA, CA199, CA724) and liver cancer diagnosis (related biomarkers: CEA, CA199, AFP). The present strategy to the synthesize redox species could be easily extended to other polymers such as polypyrrole derivatives and polythiophene derivatives. This would be of great significance in the electrochemical detection of more analytes.

Combination of multiple tumor biomarkers detection is considered to be effective ideal tool for cancer screen, diagnosis and monitoring^1^,^2^,^3^. To confirm a kind of cancer, methods that enable rapid, sensitive, reliable, inexpensive and simultaneous detection of multiple related biomarkers are highly desirable, particularly considering the large-scale explosive cancer screening^3^. Simultaneous multianalyte immunoassays (SMIAs), which can quantitatively detect multiple proteins in a signal assay is considered to be an ideal tool in analytical field^4^. Among these SMIAs, microchip capillary electrophoresis and suspension array technology have attracted great attention in the simultaneous detection of multiple biological proteins because they are high efficiency and regent saving^5^,^6^,^7^,^8^. In general, complex apparatus are commonly needed, which can increase the detection cost to some extent^9^.

Electrochemical immunoassay is a rather sensitive, and reliable method which is low-cost^10^,^11^,^12^. Recent years, a great deal of researches have been conducted on electrochemical immunoassay based on redox probes for the simultaneous detection of multiple biomarkers^13^,^14^,^15^,^16^,^17^,^18^,^19^,^20^. Among these multiplexed immunosensors, electrochemical redox specie, one for a kind of biomarker, plays a key role for achieving multiple biomarkers detection on one electrode. To date, the commonly used electrochemical redox species are dyes such as thionine, toluidine blue, methylene blue and heavy metal ions such as Cu^2+^, Cd^2+^, Pb^2+^. These redox species can be easily used for two biomarkers detection. However, the dyes can hardly produce three and more kinds of distinguishable signals in a single run, which hinders the achievement of three and more biomarkers detection. Moreover, they lead to desorption problems in case that nonchemical bonds were used for dyes fixation. The metal ions suffer from some matters such as environmentally unfriendly issues and desorption problems^17^. Therefore, novel electrochemical redox species are essentially needed for triple and more biomarkers detection.

Recently, some efficient works have been conducted for the synthesis of new electrochemical redox species^21^,^22^. Yuan’s group designed a novel electrochemical redox molecule 3,4,9,10-perylenetetracarboxylic acid/o-phenylenediamine for the detection of thrombin^22^. Polyaniline derivatives is another kind of novel electrochemical redox species which have great potential applications for multiplexed electrochemical immunosensors^23^,^24^. Aniline and its derivative are easy to be polymerized by using noble metal salt as chemical oxidating agents^25^. In principle, simultaneous detection of more biomarkers on one electrode is bad for enhancing the sensitivity and lowering the detection limit^20^. Thus, for signal amplification, good electron transfer ability and electrocatalytic ability are indispensable for designing new electrochemical redox species to enhance the sensitivity of immunosensors^21^,^22^.

It has been reported that Au/Pd alloy nanoparticles exhibited higher catalytic performance compared to monometallic Pd or Au, and the bimetallic nanoparticles were desirable nanomaterials for signal amplification in electrochemical immunoassay^26^,^27^,^28^,^29^,^30^. To this end, we used dual oxidating agents HAuCl_4_ and Na_2_PdCl_4_ to polymerize aniline derivatives and synthesized four kinds of novel redox-active nanocomposites containing Au/Pd with high H_2_O_2_ electrocatalytic ability. These composites exhibited distinguishable electrochemical signals and were used as electrochemical immunoprobes for simultaneous detection of CEA, carbohydrate antigen 19-9 (CA199), carbohydrate antigen 72-4 (CA724) and AFP in single run. The proposed method was successfully applied to detect these biomarkers in serum samples, showing its potential application in diagnosis of gastric cancer (related biomarkers: CEA, CA199, CA724) and liver cancer (related biomarkers: CEA, CA199, AFP)^31^,^32^,^33^. The present strategy to synthesize new redox species could be easily extended to the other polymers such as polypyrrole derivatives and polythiophene derivatives. This would be of great significance in electrochemical detection of more analytes.

## Results and Discussion

**Figure 1:**
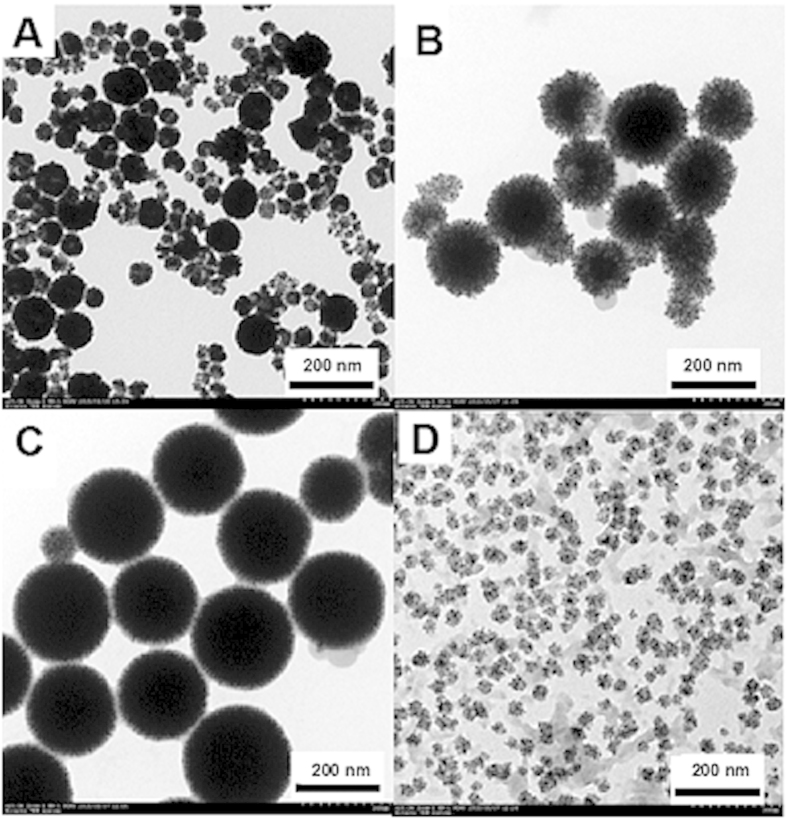
Typical TEM of PMO-Au/Pd (A); PPO-Au/Pd (B); PPP-Au/Pd (C); PTMB-Au/Pd (D).

The polyaniline derivative-Au/Pd nanocomposites were synthesized by one-step polymerizing aniline derivatives using dual oxidating agents HAuCl_4_ and Na_2_PdCl_4_ at room temperaure. The sizes of PMO-Au/Pd (Fig. 1A), PPO-Au/Pd (Fig. 1B), PPP-Au/Pd (Fig. 1C) and PTMB-Au/Pd (Fig. 1D) are ca. 100, 150, 200, and 20 nm, respectively. The elemental compositions of the PMO-Au/Pd, PPO-Au/Pd, PPP-Au/Pd, and PTMB-Au/Pd were analyzed with X ray photoelectron spectroscopy (XPS) as shown in Figure S1. The presence of Au^0^ and Pd^0^ symbol peaks indicated the successful reduction of HAuCl_4_ and Na_2_PdCl_4_ by aniline derivatives^34^,^35^.

**Figure 2:**
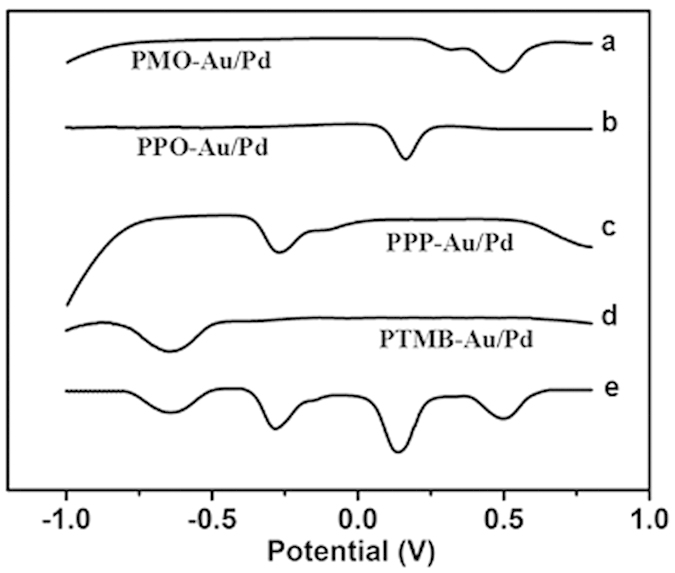
Typical SWV signals of PMO-Au/Pd (**a**), PPO-Au/Pd (**b**), PPP-Au/Pd (**c**), PTMB-Au/Pd (**d**), and a mixture of these four nanocomposites (**e**).

The electrochemical redox property of these nanocomposites was characterized by square-wave voltammetry (SWV). The PMO-Au/Pd, PPO-Au/Pd, PPP-Au/Pd and PTMB-Au/Pd exhibited distinguishable signals at −0.65 V, −0.3 V, 0.12 V, and 0.5 V in a single run as shown in Fig. 2.

To illustrate that Au/Pd in these composites plays an important role for the enhancement of electron transfer ability, electrochemical impedence spectroscopy (EIS) was used to measure the impedance of polyaniline derivative-Au/Pd and polyaniline derivatives produced by ammonium peroxydisulfate, respectively. As shown in Figure S2, smallest semicircles were observed in curves of polyaniline derivative-Au/Pd, indicating that the Au/Pd can effectively improve the electron transfer of the polyaniline derivatives and these composites exhibited better conductivity comparing with polyaniline derivatives without Au/Pd.

In order to address that the presence of bimetallic Au/Pd exhibited better electrocatalytic ability than monometal Au and Pd, amperometric i–t was conducted to investigate the H_2_O_2_ electrocatalytic ability of polyaniline derivative-Au/Pd, polyaniline derivative-Au, and polyaniline derivative-Pd, respectively. It could be observed that polyaniline derivative-Au/Pd exhibited the highest current responses followed by polyaniline derivative-Au, while polyaniline derivative-Pd diaplayed the lowest electrocatalytic ability as shown in Figure S3.

**Figure 3:**
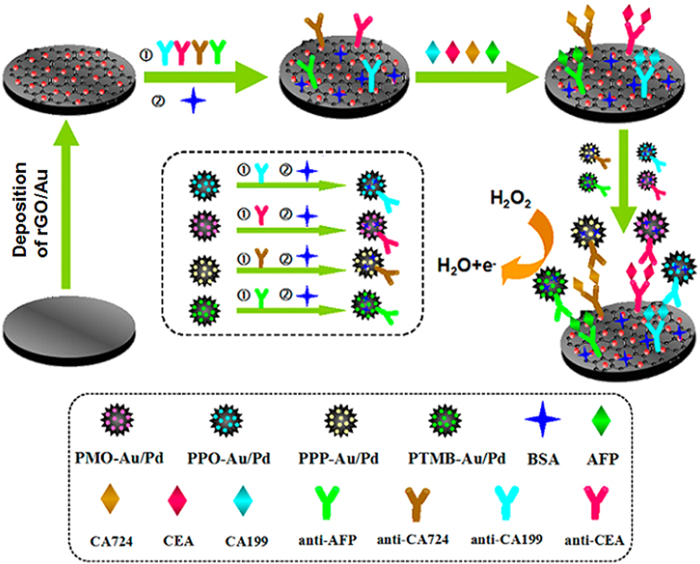
Schematic illustration of the fabrication process of the immunosensing interface .

The multianalyte immunoassay was fabricated on one electrode based on tagging strategy. The multianalyte immunoassay was fabricated on one electrode based on tagging strategy. The synthesized PMO-Au/Pd, PPO-Au/Pd, PPP-Au/Pd and PTMB-Au/Pd were used as electrochemical redox species to label anti-CEA, anti-CA199, anti-CA724, and anti-AFP, respectively. And then, the as-prepared PMO-Au/Pd-anti-CEA, PPO-Au/Pd-anti-CA199, PPP-Au/Pd-anti-CA724 and PTMB-Au/Pd-anti-AFP probes were immunoreacted with the CEA, CA199, CA724, and AFP, respectively. Multiple antigens can be simultaneously detected on one electrode through corresponding electrochemical redox species in a single electrochemical run. As is shown in Fig. 3, the glassy carbon electrode (GCE) was functionalized with reduced graphene oxide/Au (rGO/Au), followed by incubation of anti-CEA, anti-CA199, anti-CA724 and anti-AFP through Au-protein bonding and blocked by bovine serum albumin (BSA). After that, the immunsensor was incubated with a mixture solution of CEA, CA199, CA724 and AFP with different concentrations and then were dropped with polyaniline derivative-Au/Pd-antibodies. Hence, a sandwich-type immunoassay was fabricated. SWV was carried out from −1.0 V to 0.6 V and 1.5 mM H_2_O_2_ was used to amplify signals produced by PMO-Au/Pd (−0.65 V), PPO-Au/Pd (−0.3 V), PPP-Au/Pd (0.12 V) and PTMB-Au/Pd (0.5 V). Since polyaniline derivative-Au/Pd can efficiently catalyze the oxidation of H2O2, the promotion of electron transfer of these redox species and the signal amplification of this immunoassay were realized after the addition of H_2_O_2_ (Figure S4)^36^,^37^. The peak current increased with the increase of corresponding biomarker concentration. The polyaniline derivative-Au/Pd-antibody conjugates were prepared by a simple process as illustrated in Fig. 3B.

**Figure 4:**
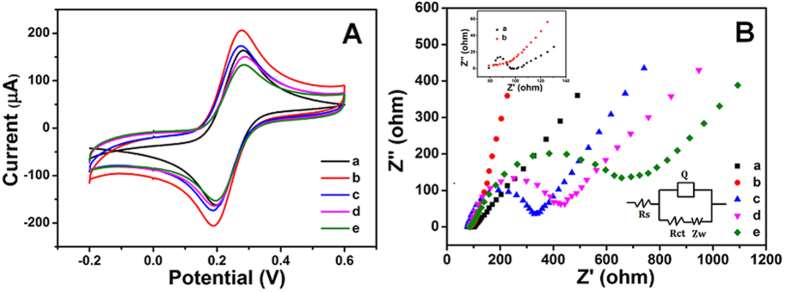
CV (A) and EIS (B) characterization of the modified procedure of electrodes in [Fe(CN)_6_]^4−/3−^.

The stepwise fabrication of the modified electrode was monitored by cyclic voltammetry (CV) and EIS. CV was conducted in 5.0 mM Fe(CN)_6_^3−/4−^ containing 0.1M KCl with a scan rate of 50 mV s^−1^ as is shown in Fig. 4A. A pair of well-defined reduction/oxidation peaks of Fe(CN)_6_^3−/4−^ were observed at the bare GCE (curve a). When the bare GCE was electrochemically deposited rGO/Au, peak current (curve b) obviously increased. The current (curve c) decreased after the rGO/Au functionalized GCE was modified with antibodies, indicating the successful attachment of antibodies on Au nanoparticles since biomolecules can retard the electron transfer^38^,^39^. Subsequently, peak current (curve d) decreased again when BSA blocked the remaining active sites and it further decreased after the adsorption of antigens (curve e).

EIS can provide further information about the modification of the electrode. Figure 4B shows the electrical impedance of the electrode in 5.0 mM Fe(CN)_6_^3−/4−^ with 0.1 M KCl. The semicircle in the high frequency region corresponds to the R_ct_, which is the most important factor reflecting the changes on the modified GCE^40^,^41^,^42^,^43^. A small semicircle can be observed for bare GCE (curve a). When rGO/Au was deposited on the GCE, almost no semicircle can be observed (curve b), implying that the rGO/Au can greatly accelerate the electron transfer. After the antibodies were incubated on the electrode, the semicircle increased remarkably (curve c). A larger semicircle diameter in curve d was observed after blocking with BSA. Semicircle was further enlarged after the immobilization of antigens on electrode (curve e). These EIS results are consistent with CV ones, revealing the successful fabrication of the immunosensing interface.

To achieve optimal performance of this immunoassay, the pH value and incubation time were optimized by incubating with target antigens including 0.1 ng mL^−1^ CEA, 0.1 U mL^−1^ CA199, 0.02 U mL^−1^ CA724 and 0.02 ng mL^−1^ AFP. As shown in Figure S5A, the current responses increased from 4.0 to 5.5 and then decreased at higher pH value. Hence, pH 5.5 was selected in this immunoassay. Figure S5B shows the current responses increased with the extension of incubation time from 15 min to 45 min and then remained constant. Therefore, the incubation time of 45 min was used in this immunoassay.

**Figure 5:**
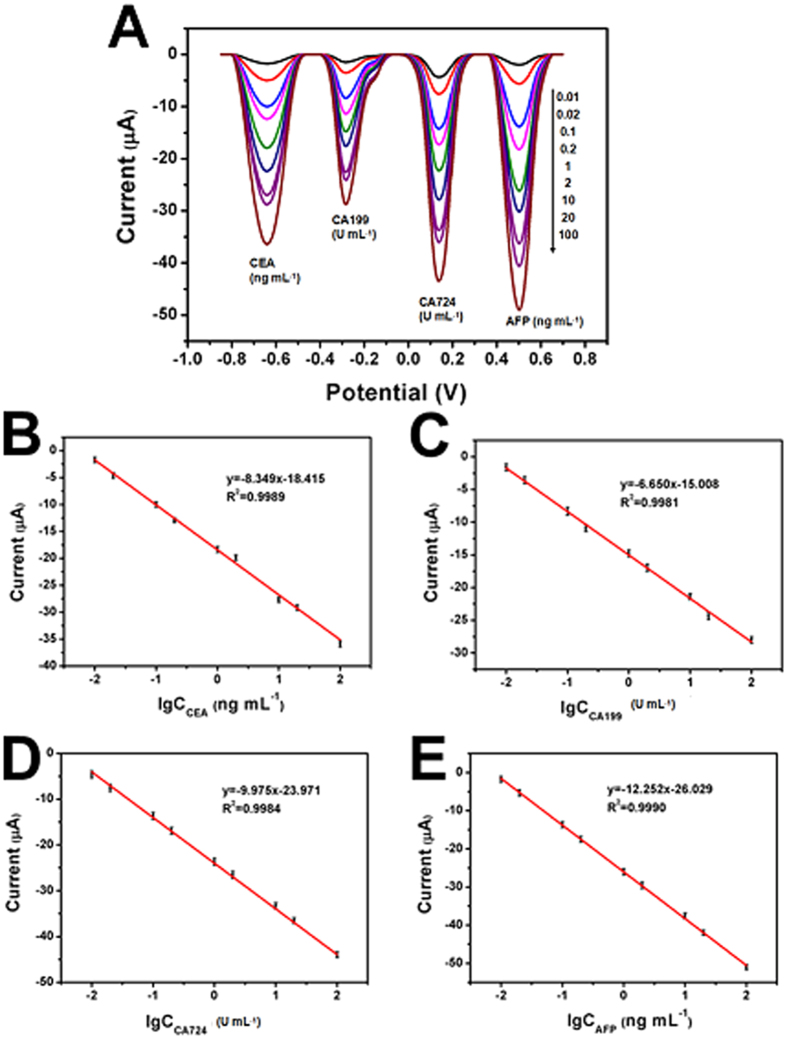
SWV responses (**A**) and calibration curves for different concentration of CEA (**B**), CA199 (**C**), CA724 (D), and AFP (E) in PBS (pH 5.5) with 1.5 mM H_2_O_2_.

Under the optimal conditions, a series of immunoassays of antigens were conducted using standard antigen solution. With the increase of antigen concentration, the peak current at −0.65 V (PMO-Au/Pd), −0.3 V (PPO-Au/Pd), 0.12 V (PPP-Au/Pd), and 0.5 V (PTMB-Au/Pd) increased as shown in Fig. 5A. The detection linear ranges from 0.01 to 100 ng mL^−1^ for both CEA (Fig. 5B) and AFP (Fig. 5E), and 0.01 to 100 U mL^−1^ for both CA199 (Fig. 5C) and CA724 (Fig. 5D). The detection limits reached 8.1 pg mL^−1^ for CEA, and 6.3 pg mL^−1^ for AFP, 0.0076 U mL^−1^ for CA199, 0.0069 U mL^−1^ for CA724, respectively. For blank experiment (without antigens), the current responses were 0.9 μA for PMO-Au/Pd probe, 0.4 μA for PPO-Au/Pd probe, 2.8 μA for PPP-Au/Pd probe, and 0.4 μA for PTMB-Au/Pd probe, respectively (n = 3). These results indicate that the non-specific adsorption of these composite is weak.

In order to address the reproducibility of the immunoassay, a well-modified electrode was measured in parallel for five times. For each of the standard antigen solution, concentrations of 0.002, 0.2 and 2 ng mL^−1^ or U mL^−1^ were chosen in this test. For CEA, the variation coefficient (VC) were 4.9%, 3.7%, 5.2%; for CA199, the VC were 3.5%, 4.2%, 3.9%; for CA724, the VC were 5.2%, 4.6%, 4.7%; for AFP, the VC were 3.2%, 2.8%, 3.4%. This showed that the present method possessed a good reproducibility. To investigate the stability of the immunosensing interface, 10 well-modified electrodes (all the antigen concentrations are 0.1 ng mL^−1^ or U mL^−1^) were reserved at 4 °C for 7 days, and the changes in peak currents were negligible (Figure S6), indicating that the present immunosensing interface possessed a good stability.

Possibly, UA, AA, DA and other antigens exist in human serum and interfere the testing of the target antigens. In order to test the anti-interference ability of this immunosensing interface, control experiments were conducted by adding UA (1 nM), AA (1 nM), DA (0.5 nM) and IgG (1 ng mL^−1^) to a mixture of antigens with final concentration 0.1 ng mL^−1^ for both CEA and AFP, 0.06 U mL^−1^ for CA199, 0.1 U mL^−1^ for CA724, respectively. As shown in Table S1, the current changes were less than 6%, indicating that the immunosensing interface exhibited good anti-interference ability. Comparison of the performance of the present and referenced multiplexed electrochemical immunosensors has been listed in Table S2. We can see that the present method exhibited a better analytical performance.

To validate the analytical application of the proposed method, ten cases of human blood serum samples were analyzed using ELISA and this method. The ELISA was used as a criterion and could provide a quantitative comparison. The results are summarized in Table 1 and the relative derivations are all within 0.28–8.33%.

## Conclusion

In summary, we have developed a one-pot route to synthesize four kinds of new electrochemical redox-active species based on HAuCl_4_ and Na_2_PdCl_4_ as co-oxidating agents and aniline derivatives as monomers. The synthesized PMO-Au/Pd, PPO-Au/Pd, PPP-Au/Pd, and PTMB-Au/Pd showed distinguishable electrochemical signals and excellent H_2_O_2_ electrocatalytic abilities. These nanocomposites were used as electrochemical immunoprobes in simultaneous detection of CEA, CA199, CA724, and AFP, showing wide linear ranges, low detection limit, good reproducibility and admirable consistency with ELISA in the detection of clinical serum samples. The present strategy could be easily extended to other polymers such as polypyrrole derivatives and polythiophene derivatives. This would be of great significance in the electrochemical detection of more analytes.

## Methods

### Materials

CEA, CA199, CA724, AFP and corresponding antibodies as well as human immunoglobulin G (IgG) were purchased from Shanghai Linc-Bio Science Co. Ltd (Shanghai, China). N-methyl-o-benzenediamine, N-phenyl-o-phenylenediamine, N-phenyl-p-phenylenediamine and 3,3′,5,5′-tetramethylbenzidine were obtained from Aladdin (Tianjin, China). Graphene oxide (GO) was purchased from Nanjing JCNANO Tech Co. Ltd (Nanjing, China). Ammonium peroxydisulfate, HAuCl_4_ ·xH_2_O, Na_2_PdCl_4_, uric acid (UA), ascorbic acid (AA), dopamine (DA) were obtained from Alfa Aesar (Tianjin, China). Ammonium peroxydisulfate were purchased from Sigma-Aldrich (USA). Clinical human serum samples were provided by the Capital Normal University Hospital (Beijing, China). N,N-dimethyl formamide (DMF), NaOH, NaH_2_PO_4_, Na_2_HPO_4_, KCl, K_3_Fe(CN)_6_, K_4_Fe(CN)_6_ and BSA were achieved from Beijing Chemical Reagents Company (Beijing, China). All other reagents were of analytical grade and used without any further purification. All aqueous solutions were prepared with ultrapure water (resistivity >18 MΩ).

### Apparatus

In all the procedures, the water used was purified through an Olst ultrapure K8 apparatus (Olst, Ltd., resistivity = 18.2 MΩ cm^−1^). Transmission electron microscope (TEM) was conducted on a JEOL-100CX electron microscope under 80 kV accelerating voltage. XPS was conducted using an Escalab 250 X-ray Photoelectron Spectroscope (Thermofisher, American) employing a monochromatic Al Kα radiation. Electrochemical measurements were carried out on CHI-832 electrochemical workstation (Chenhua Instruments Co., Shanghai, China). A three-electrode system was used in the experiment with a GCE (4 mm in diameter) as the working electrode, an Ag/AgCl electrode (saturated KCl) and a Pt wire electrode as reference electrode and counter-electrode, respectively.

### Synthesis of poly(N-methyl-o-benzenediamine)-Au/Pd (PMO-Au/Pd) probes

The PMO-Au/Pd were synthesized by adding 10 μL N-methyl-o-benzenediamine into 2.1 mL NaOH solution (4.76 mM) followed by addition of a mixture of 800 μL HAuCl_4_ (10 mM) and 800 μL Na_2_PdCl_4_ (10 mM) with vigorous string for 4 h. The composite was centrifuged at 12000 rpm for 8 min and was washed three times with ultrapure water. The obtained purified PMO-Au/Pd samples were redispersed into 3 mL ultrapure water for further functionalization. The PMO-Au/Pd-anti-CEA conjugates were prepared by adding 100 μL anti-CEA (1 mg mL^−1^) to 3 mL the resulting PMO-Au/Pd with periodic gentle mixing overnight. The conjugate was centrifuged and washed with ultrapure water for three times. Finally, BSA was used as blocking agent to cover the active sites. The conjugates were stored at 4 °C.

### Synthesis of poly(N-phenyl-o-phenylenediamine)-Au/Pd (PPO -Au/Pd) probes

The PPO-Au/Pd were synthesized by adding 100 μL N-phenyl-o-phenylenediamine (0.018 g mL^−1^ in ethnol) into 2.7 mL H_2_O followed by addition of a mixture of 400 μL HAuCl_4_ (10 mM) and 400 μL Na_2_PdCl_4_ (10 mM) with vigorous string for 4 h. The composite was centrifuged at 12000 rpm for 8 min and was washed three times with ultrapure water. The obtained purified PPO-Au/Pd samples were redispersed into 3 mL ultrapure water for further functionalization. The PPO-Au/Pd-anti-CA199 conjugates were prepared by adding 100 μL anti-CA199 (1 mg mL^−1^) to 3 mL the resulting PPO-Au/Pd with periodic gentle mixing overnight. The conjugate was centrifuged and washed with ultrapure water for three times. Finally, BSA was used as blocking agent to cover the active sites. The conjugates were stored at 4 °C.

### Synthesis of poly(N-phenyl-p-phenylenediamine)-Au/Pd (PPP-Au/Pd) probes

The PPP-Au/Pd were synthesized by adding 9 mg N-phenyl-p-phenylenediamine into 2 mL DMF followed by addition of a mixture of 200 μL HAuCl_4_ (10 mM) and 200 μL Na_2_PdCl_4_ (10 mM) with vigorous string for 4 h. The composite was centrifuged at 12000 rpm for 8 min and was washed three times with ultrapure water. The obtained purified PPP-Au/Pd samples were redispersed into 1 mL ultrapure water for further functionalization. The PPP-Au/Pd-anti-CA724 conjugates were prepared by adding 50 μL anti-CA724 (1 mg mL^−1^) to 3 mL the resulting PPP-Au/Pd with periodic gentle mixing overnight. The conjugate was centrifuged and washed with ultrapure water for three times. Finally, BSA was used as blocking agent to cover the active sites. The conjugates were stored at 4 °C.

### Synthesis of poly(3,3’,5,5’-tetramethylbenzidine)-Au/Pd (PTMB-Au/Pd) probes

The PTMB-Au/Pd were synthesized by adding 1 mL 3,3’,5,5’-tetramethylbenzidine ethanol solution (9.28 mg mL^−1^) to 2 mL H_2_O followed by addition of a mixture of 1 mL HAuCl_4_ (10 mM) and 1 mL Na_2_PdCl_4_ (10 mM) with vigorous string for 4 h. The composite was centrifuged at 12000 rpm for 8 min and was washed three times with ultrapure water. The obtained purified PTMB-Au/Pd samples were redispersed into 1 mL ultrapure water for further functionalization. The PTMB-Au/Pd-anti-AFP conjugates were prepared by adding 100 μL anti-AFP (1 mg mL^−1^) to 3 mL the resulting PTMB-Au/Pd with periodic gentle mixing overnight. The conjugate was centrifuged and washed with ultrapure water for three times. Finally, BSA was used as blocking agent to cover the active sites. The conjugates were stored at 4 °C.

### Fabrication of immunosensor

The electrode was functionalized with reduced graphene oxide/Au (rGO/Au) according to the literatures^20^,^34^,^44^. Prior to the functionalization procedure, the GCE was first polished with 0.3 nm and 0.5 nm alumina slurry respectively to get a mirror-like surface, sonicated with ultrapure water and dried at 37 °C. After pretreatment, GCE was submerged in a solution containing 1 mg mL^−1^ GO and 100 μM HAuCl_4_ in the presence of magnetic string and N_2_ bubbling for 1 min. After this time, CV was carried out between −1.5 V and 0.0 V with a scan rate of 50 mV s^−1^ for five potential cycles. As a result, homogenous rGO/Au multilayer films were formed on the bare GCE and were used as substrate in this immunoassay. The obtained rGO/Au-functionalized substrate was then placed in a solution containing anti-CEA, anti-CA199, anti-CA724 and anti-AFP with each concentration of 200 μg mL^−1^ for a period of 12 h and rinsed with PBS. Then, the substrate was treated with 1% BSA , followed by incubated in a mixture of CEA, CA199, CA724, and AFP with each concentration of 0.01, 0.02, 0.1, 0.2, 1, 2, 10, 20, and 100 ng mL^−1^ or U mL^−1^ at 37 °C for 45 min. Prior to immobilization procedure, the resulted polyaniline derivative-Au/Pd-antibodies conjugates were mixed with 1% BSA for 45 min to block the remaining active sites, respectively, and then were centrifuged and washed with ultrapure water for three times. Next, the as-prepared polyaniline derivative-Au/Pd-antibodies conjugates were mixed in a suitable relations (1:2:4:1) with gentle stirring for 45 min. Finally, 20 μL of the mixture was dropped on the antigens-antibodies-rGO/Au-modified substrate followed by incubation at 37 °C for 45 min and then rinsed with PBS.

### Human serum samples analysis

Prior to the human serum samples analysis, the serum samples were diluted with five times with ultrapure water. And then, 80 μL the diluted human serum samples was dropped on the antigen modified rGO/Au-functionalized electrode, and incubated at 37 °C for 45 min. After the electrode was wash by ultrapure water, 20 μL the mixed polyaniline derivative-Au/Pd-antibodies conjugate was incubated on the above electrode at 37 °C for 45 min.

### Electrochemical measurement

All electrochemical measurements were carried out at room temperature in 0.1 M PBS (pH 5.5). The immunoassay was acted as work electrode and SWV was conducted from −1.0 V to 0.6 V with pulse amplitude of 25 mV and a frequency of 15 HZ (the sensitivity is 10^3^). In the curves of SWV, four distinguishable oxidation peaks appeared and each peak indicated one target antigen (i.e. CEA at −0.65 V, CA199 at −0.3 V, CA724 at 0.12 V, AFP at 0.5 V).

## Additional Information

**How to cite this article**: Wang, L. *et al.* Novel electrochemical redox-active species: one-step synthesis of polyaniline derivative-Au/Pd and its application for multiplexed immunoassay. *Sci. Rep.* **5**, 16855; doi: 10.1038/srep16855 (2015).

## Supplementary Material

Supplementary Information

## Figures and Tables

**Table 1:** Determination of CEA, CA199, CA724, and AFP in serum samples (n = 3).

Sample No.		1	2	3	4	5	6	7	8	9	10
This work	CEA (ng mL^−1^)	1.36	1.22	0.66	2.31	0.77	1.11	5.29	8.26	1.10	0.60
	CA199 (U mL^−1^)	7.29	7.99	9.11	9.66	7.20	0.39	3.68	7.79	9.54	8.12
	CA724 (U mL^−1^)	1.31	0.85	1.41	0.17	1.10	0.80	1.10	2.00	0.31	0.99
	AFP (ng mL^−1^)	0.15	0.19	0.68	0.15	0.22	4.49	0.50	0.84	0.20	0.63
ELISA	CEA (ng mL^−1^)	1.33	1.26	0.62	2.27	0.80	1.16	5.32	8.29	1.12	0.61
	CA199(U mL^−1^)	7.22	7.95	9.15	9.69	7.18	0.40	3.70	7.75	9.50	8.05
	CA724 (U mL^−1^)	1.35	0.89	1.38	0.18	1.06	0.77	1.08	2.04	0.36	0.96
	AFP (ng mL^−1^)	0.16	0.18	0.73	0.14	0.24	4.52	0.53	0.89	0.21	0.65
Relative error (%)	CEA	−3.17	−3.17	6.45	1.76	−3.75	−4.31	−0.56	−0.36	−1.79	−1.64
	CA199	0.50	0.50	−0.44	−0.31	0.28	−2.50	−0.54	0.52	0.42	0.90
	CA724	−2.96	−4.49	2.17	−5.56	3.78	3.90	1.85	−1.96	−2.78	3.13
	AFP	−6.25	5.56	−6.85	7.14	−8.33	−0.63	−5.66	−5.62	−4.76	−3.08
