# Supplementary material for: Novel electrochemical redox-active species: one-step synthesis of polyaniline derivative-Au/Pd and its application for multiplexed immunoassay
Source: Sci Rep. 2015 Nov 18;5:16855. doi: 10.1038/srep16855 (PMC4649611; doi:10.1038/srep16855)
Supplement: Supplementary Information [file srep16855-s1.doc]

Supplementary material

**Novel electrochemical redox-active species: one-step synthesis of polyaniline derivative-Au/Pd and its application for multiplexed immunoassay**

Liyuan Wang, Feng Feng, Zhanfang Ma*

Department of Chemistry, Capital Normal University, Beijing 100048, China

The overview spectra in Figure S1A-D revealed that C, N, Au, Pd atoms are present in these composites. The Au 4f doublet for PMO-Au/Pd (83.7 eV and 87.5 eV), PPO-Au/Pd (83.8 eV and 87.5 eV), PPP-Au/Pd (83.9 eV and 87.6 eV) and PTMB-Au/Pd (83.7 eV and 87.5 eV) in Figure S1E-H, respectively, are consistent with Au^0^ state, indicating HAuCl_4_ was successfully reduced to Au^0^. The Pd 3d doublet for PMO-Au/Pd (334.5 eV and 339.9 eV), PPO-Au/Pd (334.8 eV and 340.3 eV), PPP-Au/Pd (334.1 eV and 339.5 eV) and PTMB-Au/Pd (334.9 eV and 340.5 eV) in Figure S1I-L, respectively, are consistent with Pd^0^ state, indicating the successful reduction of Na_2_PdCl_4_ to Pd^0^.


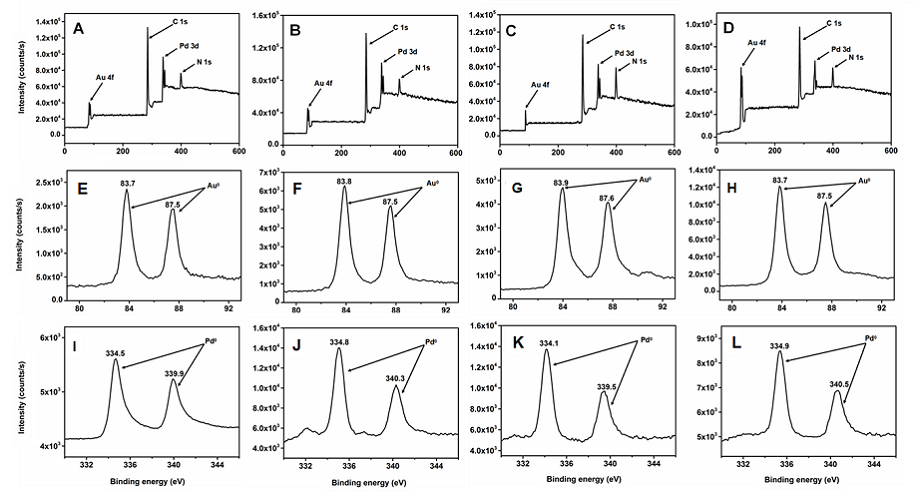


**Figure S1.** XPS spectrum of PMO-Au/Pd (**A**), PPO-Au/Pd (**B**), PPP-Au/Pd (**C**) and PTMB-Au/Pd (**D**); high-resolution XPS of Au 4f spectrum of PMO-Au/Pd (**E**), PPO-Au/Pd (**F**), PPP-Au/Pd (**G**), PTMB-Au/Pd (**H**); high-resolution XPS of Pd 3p spectrum of PMO-Au/Pd (**I**), PPO-Au/Pd (**J**), PPP-Au/Pd (**K**), and PTMB-Au/Pd (**L**).


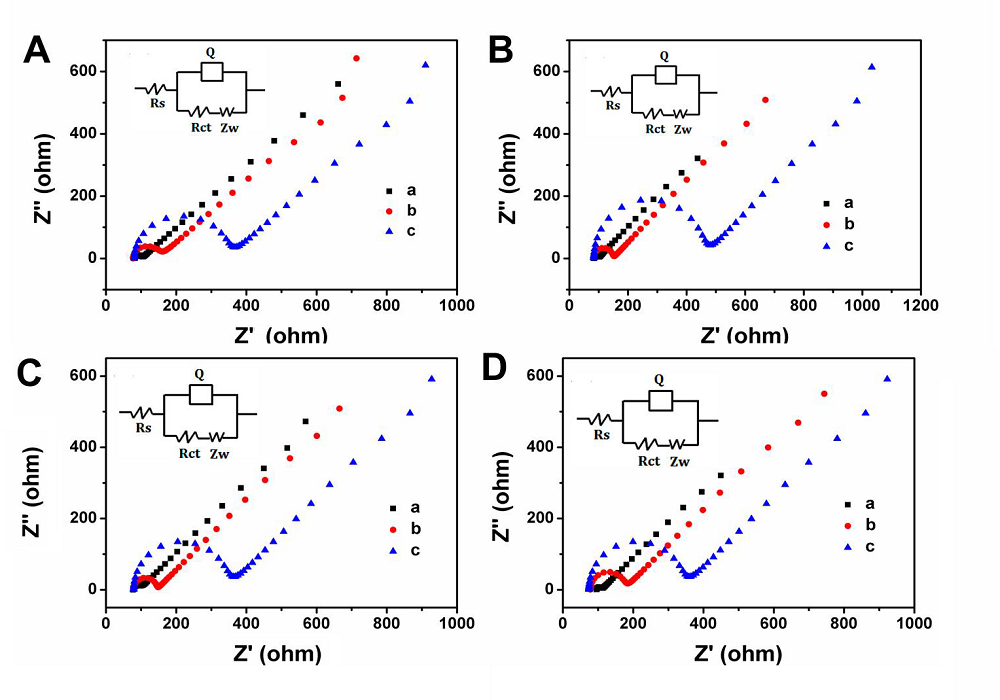


**Figure S2.** EIS of GCE modified with PMO-Au/Pd (curve **a**), bare GCE (curve **b**), PMO (curve **c**) (**A**); PPO-Au/Pd (curve **a**), bare GCE (curve **b**), PPO (curve **c**) (**B**); PPP-Au/Pd (curve **a**), bare GCE (curve **b**), PPP (curve **c**) (**C**) and PTMB-Au/Pd (curve **a**), bare GCE (curve **b**), PTMB (curve **c**) (**D**).


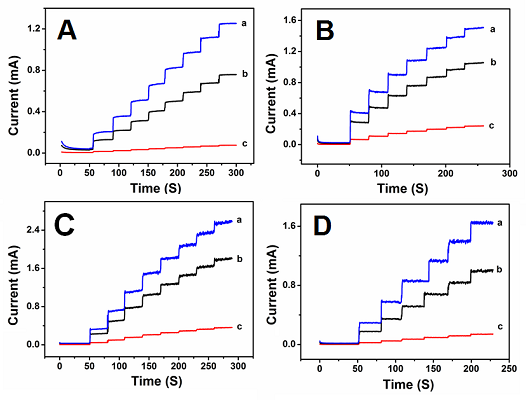


**Figure S3.** Ameperometic response of PMO-Au/Pd (curve **a**), PMO-Au (curve **b**), PMO-Pd (curve **c**) (**A**); PPO-Au/Pd (curve **a**), PPO-Au (curve **b**), PPO-Pd (curve **c**) (**B**); PPP-Au/Pd (curve **a**), PPP-Au (curve **b**), PPP-Pd (curve **c**) (**C**) and PTMB-Au/Pd (curve **a**), PTMB-Au (curve **b**), PTMB-Pd (curve c) (D) for successive additions of 5 mM H_2_O_2_.


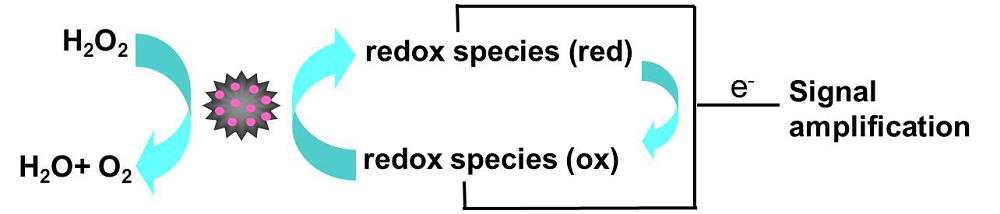


**Figure S4.** Mechanism of signal amplification by electrocatalytic process.

**
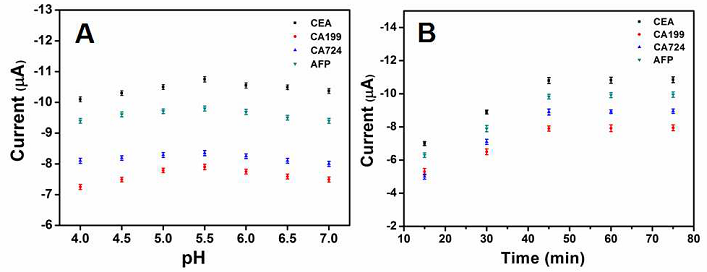
**

**Figure S5.** Effect of pH of electrolyte (**A**) and incubation time (**B**) on the SWV currents.


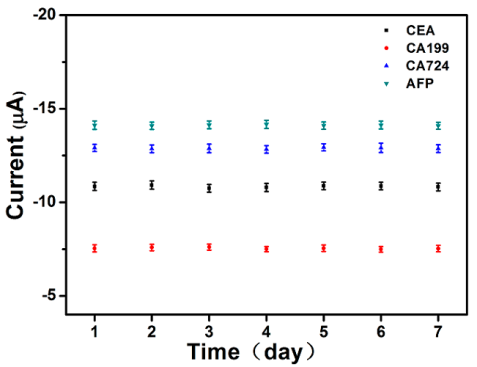


**Figure S6.** The stability of the immunosensing interface for the detection of 0.1 ng mL^-1^ CEA and AFP, 0.1 U mL^-1^ CA199 and CA724.

**Table S1.** Anti-reference ability of the immunoassay.

| Interferents | Blank | UA | AA | DA | IgG |
| --- | --- | --- | --- | --- | --- |
| CEA (μA) | 11.6 | 11.1 | 11.3 | 12.1 | 11.2 |
| Current changes of CEA caused by interferents (%) | - | -4.31 | -2.59 | 4.31 | -3.45 |
| CA199 (μA) | 8.7 | 8.2 | 8.9 | 9.1 | 8.3 |
| Current changes of CA199 caused by interferents (%) | - | -5.75 | 2.30 | 4.60 | 4.60 |
| CA724 (μA) | 14.8 | 15.5 | 14.1 | 14.4 | 15.4 |
| Current changes of CA724 caused by interferents (%) | - | 4.73 | -4.73 | -2.70 | 4.05 |
| AFP (μA) | 15.1 | 14.4 | 15.8 | 15.9 | 14.5 |
| Current changes of AFP caused by interferents (%) | - | -4.64 | 4.63 | 5.30 | -3.97 |

**Table S2.** Comparison of the performance of the present and referenced multiplexed electrochemical immunosensors.

| Detection targets | Detection range | Detection limit | Reproductivity | Stability | Reference |
| --- | --- | --- | --- | --- | --- |
| CEA, AFP | 0.5-45 ng mL^-1^*^a^*  1-90 ng mL^-1^*^b^* | 0.2 ng mL^-1^*^a^*  0.5 ng mL^-1^*^b^* | 3.9%*^a^*  4.3 %*^b^* | 3.4%*^a^*; 5.3%*^b^* (4 weeks) | [1] |
| CA125, CA199 | 0-24 U mL^-1^ *^c^* | 0.2 U mL^-1^*^c^* | > 7%*^c^* | 5.1%*^c^*  (4 weeks) | [2] |
| CEA, NSE, CYFRA21-1 | 0.01-100 ng mL^-1^*^a^* | 6.3 pg mL^-1^*^a^* | 3.54%*^a^* | > 5%*^a^*  (1 week) | [3] |
| CA125,  CA153, CA199 | 0.01-60 U mL^-1^ *^c^* | 0.002 U mL^-1^*^c^* | > 11%*^c^* | 13.8 %*^c^*  (4 weeks) | [4] |
| CEA, CA153, CA125, CA199 | 0.16-9.2 ng mL^-1^*^a^*  0.16-15 U mL^-1^*^c^* | 0.04 ng mL^-1^*^a^*  0.10 U mL^-1^*^c^* | - | > 2% *^a, c^*  (10 min) | [5] |
| CEA, AFP, CA199, CA724 | 0.01-100 ng mL^-1^*^a, b^*  0.01-100 U mL^-1^ *^c ,d^* | 8.1 pg mL^-1^*^a^*  6.3 pg mL^-1^*^b^*  0.0076 U mL^-1^*^c^*  0.0069 U mL^-1^*^d^* | > 5.2% *^a, b^;*  > 4.2% *^c^*  > 3.4% *^d^* | 2.1% *^a^*  1.9% *^b^*  2.5% *^c^*  2.3% *^d^*  (1 week) | This work |

*^a^* Mean the result for CEA; *^b^* mean the result for AFP; *^C^* mean the result for CA199; *^d^* mean the result for CA724.

1. Lin, J. H., Wei, Z. J., Mao, C. M., A label-free immunosensor based on modified mesoporous silica for simultaneous determination of tumor markers. *Biosens. Bioelectron.* **29**, 40-45 (2011).
2. Wu, J., Zhang, Z. J., Fu, Z. F., Ju, H. X., A disposable two-throughput electrochemical immunosensor chip for simultaneous multianalyte determination of tumor markers. *Biosens. Bioelectron.* **23**, 114-120 (2007).
3. Wang, L. Y., Liu, N., Ma, Z. F., Novel gold-decorated polyaniline derivatives as redox-active species for simultaneous detection of three biomarkers of lung cancer. *J. Mater. Chem. B* **3**, 2867-2872 (2015).
4. Tang, D. P., Hou, L., Niessner, R., Xu, M. D., Gao, Z. Q., Knopp, D. M., Multiplexed electrochemical immunoassay of biomarkers using metal sulfide quantum dot nanolabels and trifunctionalized magnetic beads. *Biosens. Bioelectron.* **46**, 37-43 (2013).
5. Wu, J., Yan, Y. T., Fan, F., Ju, H. X., Electric field-driven strategy for multiplexed detection of protein biomarkers using a disposable reagentless electrochemical immunosensor array. Anal. Chem. **80**, 6072-6077 (2008).
